# HIV Testing and Risks of Sexual Behavior among HIV-Negative Men Who Have Sex with Men in Ningbo, China

**DOI:** 10.3390/ijerph17041322

**Published:** 2020-02-19

**Authors:** Haibo Jiang, Hang Hong, Hongjun Dong, Jun Jiang, Lin He

**Affiliations:** 1 Ningbo Municipal Center for Disease Control and Prevention, Ningbo 315010, China; jianghaibo212@163.com (H.J.); hongh@nbcdc.org.cn (H.H.); 2 Zhejiang Provincial Center for Disease Control and Prevention, Hangzhou 310051, China; jjiang@cdc.zj.cn

**Keywords:** HIV-negative, men who have sex with men, testing, behavior

## Abstract

Human immunodeficiency virus (HIV) testing is confirmed as a preventive strategy for HIV control. However, the testing rate and risk behaviors of HIV-negative men who have sex with men (MSM) remain unclear. We aimed to examine factors associated with HIV testing and high-risk behaviors among HIV-negative MSM. From July 2016 to June 2017, participants were recruited by snowball sampling from WeChat groups, bars, and other venues. HIV testing was performed to exclude HIV-positive MSM. Face-to-face questionnaires regarding HIV testing and high-risk behaviors were conducted; 988 MSM were included, and 57.1% of participants underwent HIV testing in the past year. The proportion of high-risk behaviors was 49.9%. Factors associated with HIV testing were bisexual orientation, substance use to adjust psychiatric disorders, and receiving acquired immune deficiency syndrome (AIDS) interventions. Being married, bisexual orientation, and receiving AIDS interventions were risk factors for high-risk sexual behaviors, while college or higher degree was a protective factor. We determined that HIV transmission factors are widespread, and the rate of HIV testing is relatively low. Attention should be given to marital status, using substances to adjust psychiatric disorders, or bisexual HIV-negative MSM, and AIDS interventions should be strengthened to promote HIV testing and reduce high-risk behaviors.

## 1. Introduction

In 2014, the Joint United Nations Program on human immunodeficiency virus (HIV)/acquired immune deficiency syndrome (AIDS) proposed the vision of “ending AIDS in 2030” and achieving “three 90%” prevention and treatment goals in 2020 [1]. These targets include 90% of individuals living with HIV being aware of their HIV infection, 90% of those receiving antiretroviral treatment (ART), and 90% of people receiving ART having successful suppression of viral load. In 2017, China also used the “three 90%” as the work target of the “13th Five-Year Plan of Action for China’s Containment and Prevention of AIDS” [2]. At present, treatment as a prevention strategy has been confirmed [3,4]; the last two 90% targets would not be difficult to implement, but the first 90% target faces enormous challenges. HIV testing is confirmed as a key HIV prevention strategy. Early HIV detection can not only expand treatment for people living with HIV/AIDS (PLWHA), but also strengthen interventions for individuals who have tested positive for HIV [5], reducing the proportion of high-risk behaviors and risk of HIV infection among uninfected individuals [6]. According to the estimated epidemic situation in Zhejiang Province in 2016 [7], the proportion of PLWHA diagnosed at the end of 2016 was only 71.3%, and the diagnosis rate among men who have sex with men (MSM) was 62.0%, which was lower than the diagnosis rate in individuals who acquired HIV infection through heterosexual contact, injecting drug use, or mother-to-child transmission.

The HIV infection rate in the MSM population is growing rapidly in China mainly due to multiple sexual partners and high-risk sexual behaviors, and MSM are an important population of HIV infection and transmission in China [8,9]. In Western countries, the HIV prevalence rate and estimated morbidity rate of young MSM are also high, and they are also a key population of HIV transmission [10]. Globally, even in countries or regions with high HIV prevalence, the HIV testing rate among MSM remains low (1.6–41.7%) [11], and the annual HIV testing rate among MSM in China is also low (44.6–69%) [12,13,14]. The key work is to promote HIV testing and reduce high-risk behaviors in MSM to finally achieve the goal of the first 90% target.

In 2015, the World Health Organization recommended AIDS treatment as a prevention strategy [15]. In 2016, China began to implement the “treatment as prevention” strategy [16]. HIV-positive individuals are targeted, intervened, and arranged for treatment after HIV confirmation; thus, their contagiousness will be greatly reduced [3,17]. Relevant studies have shown that HIV-positive individuals change their high-risk behaviors after confirmation [18,19,20]. Some studies have shown that compared with HIV-negative individuals, PLWHA are more likely to develop unprotected anal sex behavior (UAI) [21,22]. Meanwhile, other studies have suggested that a small number of HIV-negative MSM may significantly increase community-level HIV transmission because of reduced willingness to undergo HIV testing [23].

As a susceptible population for HIV, data on the AIDS-related behavior among HIV-negative MSM are lacking. Although HIV testing is increasingly popular among MSM, little is known about the patterns of behaviors of HIV-negative MSM. From July 2016 to June 2017, we established a cohort of 988 HIV-negative MSM to explore new HIV infections and effective intervention methods in Ningbo, China. The baseline survey data were used to describe the patterns of behavior and examine the factors associated with HIV testing and risk behaviors.

## 2. Materials and Methods

### 2.1. Design and Sampling Methods

This was a cross-sectional study conducted in Ningbo City, China, between July 1, 2016, and June 30, 2017.

We used snowball sampling methods to recruit respondents; MSM were recruited from Blued (an app for the gay community), QQ/WeChat groups, voluntary counseling and testing clinics, bathrooms, bars, and other types of venues. The initial participants were asked to recruit partners or peers to participate in the survey. After the participants completed the study questionnaires and underwent HIV testing, they were provided with recruitment coupons to incentivize other participants. With these incentives, the participants then recruited MSM peers from their social network to participate in the study. Without knowledge of the rate of HIV testing among HIV-negative MSM in Ningbo, we referred to studies conducted in other regions of China to determine the sample size. These studies showed that the HIV testing rate ranged from 50% to 60%. *P*-values < 0.05 and β = 0.1 were considered statistically significant. Thus, the estimated minimum sample size required for this study was 514. The sample size was calculated online (http://powerandsamplesize.com).

### 2.2. Study Participants

We included MSM who met the following criteria: 1) at least 18 years of age; 2) had anal sex with men in the last 3 months; 3) HIV-negative or unknown status, and 4) provided informed consent to participate in the study. MSM were excluded from the study if their results of screening tests were positive; they were impaired and could not clearly understand and answer the questions from the investigation questionnaire because of excessive alcohol consumption, poisoning, or other causes, or because they had a mental illness or mental retardation; they did not fully understand the process of informed consent or did not give informed consent; they did not agree to accept the questionnaire survey and/or serological survey; or they refused to take part in the study for any other reasons.

### 2.3. Questionnaire, Data Collection, and Dependent Variable Definition

A face-to-face questionnaire survey was conducted, which included questions about socio-demographic characteristics like age, marital status, education, income, sexual orientation, registered residence and living time in Ningbo, sexual behaviors, sexual partner networks in the past 3 months, history of HIV testing, and history of counseling. After the interview, all the participants were tested for HIV, free of charge. Combining expert consultation opinions, panel discussion opinions, previous relevant literature, and pre-survey trials, self-developed questionnaires were used after being reviewed by experts in related fields. All questionnaires were completed by an investigator who was a unified professional trained at county or district CDCs in Ningbo.

HIV testing was defined as MSM who ever underwent HIV-1 testing in the past year. High-risk sexual behaviors meant that MSM engaged in sexual behaviors without condom use in the past 3 months; MSM who engaged in sexual behaviors with condom use or had not been sexual were defined as non-high-risk behaviors.

### 2.4. HIV Testing

A venous blood specimen (5 mL) was drawn from each participant following the interview. The HIV-1 screening test was performed using an enzyme-linked immunosorbent assay (ELISA; Anti-HIV ELISA Kit, Zhuhai Livzon Diagnostics Inc., China), and if the result was positive, the specimen was retested using the same ELISA kit as well as another ELISA kit (Anti-HIV ELISA Kit, Beijing Wantai Biological Pharmacy Enterprise Co. Ltd., China).

### 2.5. Statistical Analysis

Data were entered into EpiData version 3.1 (http://www.epidata.dk/) via double entry. After the data were cleaned and verified, statistical analysis was performed using SPSS version 23.0. For descriptive analyses, categorical variables are presented as frequencies and proportions, and continuous variables are presented as medians and interquartile ranges (IQRs). Differences in the general demographic characteristics were calculated using Student’s t-tests, chi-squared (χ^2^) tests, Fisher’s exact test, and Kruskal–Wallis tests. We determined the mean (standard deviation [SD]) and median (IQR) differences. Factors from univariate analysis with *P*-values < 0.10 and/or those previously shown to be associated with differences in socio-demographic characteristics were included in the multivariate regression models, which were used to calculate adjusted odds ratios (AORs) and their 95% confidence intervals (CIs). The primary outcomes of interest were HIV testing in the past 12 months, bisexual orientation, substance use to adjust psychiatric disorders, and having undergone AIDS interventions in the past 3 months. *P*-values < 0.05 and β = 0.1 were considered statistically significant.

### 2.6. Ethical Considerations

The study was approved by the ethical review board of Ningbo Municipal Center for Disease Control and Prevention. Participants were not harmed by their participation in the study and their data remained confidential. Written informed consent was signed by all participants during the survey. The charge for HIV testing was free. Participants received 50 RMB (approximately 7.2 US$) for their participation in the survey. Participants with positive results in the HIV testing were informed and counseled by the staff of local county or district CDCs and received the necessary referral services. This informing and counseling was provided at the same location as the interviews.

## 3. Results

In total, 988 HIV-negative MSM were recruited to complete the survey, and 2 MSM were excluded because their screening test results were positive. The total response rate of this study was 100%. The median age of the respondents was 29.8 ± 8.4 years. Moreover, 602 (60.9%) MSM were single and 346 (35.0%) were married, 978 (99.0%) were of Han nationality, 398 (40.3%) had a college or higher degree, and the monthly income of 480 (50.5%) participants was greater than 4000 RMB. The majority of MSM were homosexual (*n* = 609, 61.6%) or bisexual (*n* = 313, 31.7%), registered residents of Zhejiang province (*n* = 528, 53.4%), recruited through the Internet (*n* = 529, 53.5%), and living in Ningbo for more than 24 months (*n* = 724, 73.3%, Table 1).

Of the 988 recruited MSM, 57.1% (564/988) had undergone HIV testing in the past year. The proportion of high-risk sexual behaviors was 49.9%. Among all respondents, 153 (15.5%) engaged in heterosexual high-risk sexual behaviors, 432 (43.7%) engaged in homosexual high-risk behaviors, 92 (9.3%) engaged in high-risk sexual behaviors with both male and female partners, 57 (5.8%) individuals had anal unprotected sex after drinking alcohol, 8 (0.8%) had anal sex without condom use after using drugs, and 20 (2.0%) had group anal sex in the past 3 months. Furthermore, 31 (3.1%) had been diagnosed with sexually transmitted diseases (STD/S) in the last 3 months, and 430 (43.5%) had received AIDS interventions in the past 3 months. The awareness rate of AIDS knowledge was 91.7% (906/988); 896 (90.7%) individuals believed that the current HIV epidemic situation was not serious or average, and 441 (44.6%) individuals used substances to adjust psychiatric disorders (Table 2).

There were significant differences in the age (*P* = 0.016), sample source (*P* = 0.014), monthly income (*P* = 0.005), and sexual orientation (*P* = 0.008) between HIV-negative MSM who had undergone HIV testing in the past year and those who had not. There were no significant differences in the other demographic characteristics investigated (*P* > 0.05). Among AIDS-related knowledge and behavioral variables, there were significant differences in the cognition of the HIV epidemic among MSM (*P* < 0.001), drug use to adjust psychiatric disorders (*P* < 0.001), diagnosis with STD/S in the last 3 months (*P* = 0.020), and receiving AIDS interventions in the past 3 months (*P* < 0.001) between HIV-negative MSM who had undergone HIV testing in the past year and those who had not. There were no significant differences in the other behavioral characteristics investigated (*P* > 0.05).

Univariate analysis showed that bisexual orientation (odds ratio [OR] 0.65, 95% CI 0.50–0.86), substance use to adjust psychiatric disorders in the past week (OR 1.88, 95% CI 1.45–2.44), and receiving AIDS interventions in the past 3 months (OR 4.44, 95% CI 3.35–5.87) were associated with HIV testing in the past year (Table 3). Multivariate logistic regression analysis showed that bisexual orientation (AOR 0.57, 95% CI 0.42–0.78), substance use to adjust psychiatric disorders (AOR 1.39, 95 CI 1.04–1.85), and receiving AIDS interventions in the past 3 months (AOR 4.03, 95 CI 3.00–5.42) were factors associated with HIV testing in the past year (Table 3).

Univariate analysis showed that being married (OR 2.16, 95% CI 1.65–2.83), having a college or higher degree (OR 0.47, 95% CI 0.32–0.65), bisexual orientation (OR 2.46, 95% CI 1.86–3.26), and receiving AIDS interventions in the past 3 months (OR 1.54, 95% CI 1.20–2.0) were associated with high-risk sexual behaviors in the past 3 months (Table 4). Multivariate logistic regression analysis also confirmed that being married (AOR 1.72, 95% CI 1.15–2.58), having bisexual orientation (AOR 2.13, 95% CI 1.54–2.95), and receiving AIDS interventions in the past 3 months (AOR 1.65, 95% CI 1.25–2.20) were risk factors for high-risk sexual behaviors in the past 3 months. In addition, it also showed that a college or higher degree (AOR 0.52, 95% CI 0.35–0.77) was a protective factor for high-risk sexual behaviors in the past 3 months (Table 4).

## 4. Discussion

Our study revealed that the HIV-negative MSM population had a low rate of HIV testing, which poses a major challenge to achieving the target of 90% of patients infected with HIV being diagnosed in 2020. Meanwhile, HIV-negative MSM engaged in widespread high-risk sexual behaviors, including group sex and sexual intercourse after drinking or after drug use. There may still be a large risk of new HIV infection in this population. Since there is a certain proportion of heterosexual high-risk sexual behaviors and high married rate among this population, bisexual or married MSM would be the key population for HIV prevention and control in the future among HIV-negative MSM.

In addition to two MSM who were excluded from the survey, the HIV testing rate was found to be only 57.0% (564/990) in the last year, which was similar to the results of the respondent-driven sampling survey among MSM conducted by LI R et al. in Hangzhou (56.8%) [24], but lower than the proportion of detection reported in the US in 2013 (67.0%) [25]. This could be because of previous low HIV intervention coverage (43.5%) and because work to promote testing for MSM sexual partners or self-testing interventions had not been carried out in large areas. We found that HIV detection among bisexual MSM was insufficient, compared with that among homosexual MSM. It is necessary to carry out measures to promote HIV detection among HIV-negative MSM, such as sexual partner promotion test or HIV self-testing [26], especially among bisexual MSM. It was also found that there were a group of MSM (44.6%) with psychiatric disorders (e.g., annoyance, nervousness, depression, etc.), and using substances (e.g., cigarettes, alcohol, prescription psychotropic drugs, illegal drugs, etc.) to adjust their psychiatric disorders. Besides the basic traditional HIV high-risk sexual behavior interventions, joint interventions with psychiatric counseling and detection consciousness should be strengthened to improve the mental health of this population [27].

We found the awareness rate of AIDS knowledge (91.7%) among HIV-negative MSM was high, but the proportion of high-risk behaviors in the past three months was as high as 49.9%, which was lower than the rate revealed by Lin He and others who conducted a survey in Hangzhou in 2015–2016 [28]. Moreover, the proportion of high-risk homosexual behaviors (43.7%) was also lower than that in the results of US-based surveys [26,29]. Above all, the results showed that the local HIV-negative MSM had more common high-risk behaviors, which was similar to some previous research results [30,31]. We also found that the proportion of high-risk behaviors in those of bisexual orientation was higher than that in those of homosexual orientation (AOR = 2.130), but the detection awareness was insufficient (50.5%), and the proportion of marriage was higher (35%), which was similar to the results of previous investigations in China and abroad [32,33,34], which suggested that bisexual or married MSM had a risk of infection and transmission of HIV. Thus, interventions for high-risk behaviors and HIV-testing promotion for married or bisexual MSM should be strengthened.

The results also suggested that AIDS intervention can be a contributing factor to HIV testing and high-risk sexual behaviors. Since AIDS interventions includes AIDS-related knowledge education, HIV testing and high-risk behavior intervention, and 76.5% of participants who had received AIDS interventions had undergone HIV testing, it may be inferred that AIDS interventions were catalyst for HIV testing. As AIDS interventions have been proven to reduce high-risk sexual behaviors in previous research [35], the proportion of high-risk behaviors may have been reduced if the intervention effect was positive enough. However, the proportion of high-risk behaviors among those who had received AIDS intervention was relatively high (56.0%). This might be because those MSM had received AIDS intervention because of engaging in high-risk sexual behaviors before, and it may also indicate that the effect of the intervention was not satisfactory enough to reduce high-risk behaviors. Then, the quality and coverage of AIDS interventions should be improved. The role of AIDS interventions in reducing high-risk sexual behaviors will be clarified in our subsequent interventional cohort studies.

Our study had several limitations. First, this was a cross-sectional study; therefore, the causal factors associated with HIV testing and high-risk sexual behaviors could not be determined. Second, we did not use probabilistic sampling; instead, we used snowball sampling to recruit MSM. Although the MSM were recruited from a variety of settings, we could not determine the rate of HIV testing and high-risk behaviors for all MSM in Ningbo. Last, some items in the questionnaire contained some information with a large time span or sensitive information; thus, some participants might not have reported their privacy behavior. Therefore, the frequency of HIV testing and risks of sexual behaviors might have been underestimated.

## 5. Conclusions

We noted a low rate of HIV testing, high proportion of high-risk sexual behaviors, and higher rates of marriage among HIV-negative MSM in Ningbo. HIV testing was associated with bisexual orientation and substance use to adjust psychiatric disorders; being married, having bisexual orientation, or having a lower educational level tended to be associated with high-risk sexual behaviors among HIV-negative MSM. We suggest that attention should be given to marital status, the use of substance to adjust psychiatric disorders, or bisexual HIV-negative MSM, and AIDS interventions should be strengthened to promote HIV testing and reduce high-risk sexual behaviors.

## Figures and Tables

**Table 1:** Socio-demographic characteristics of HIV-negative men who have sex with men who underwent HIV testing in the past year in Ningbo, China (N = 988).

Variables	N (%)	HIV Testing		χ^2^	*P*
		Yes	No		
Age				8.290	0.016
<30	573(58.0)	318(55.5)	255(44.5)		
30–50	389(39.4)	237(60.9)	152(39.1)		
50–	26(2.6)	9(34.6)	17(65.4)		
Marital status				2.514	0.285
Single	602(60.9)	336(55.8)	266(44.2)		
Married	346(35.0)	208(60.1)	138(39.9)		
Divorced/widowed	40(4.0)	20(50.0)	20(50.0)		
Ethnicity				-	0.752
Han	978(99.0)	559(57.2)	419(42.8)		
Minority	10(1.0)	5(50.0)	5(50.0)		
Education				2.871	0.238
Junior high school and below	276(27.9)	169(61.2)	107(38.8)		
High school and junior college	314(31.8)	177(56.4)	137(43.6)		
College or higher degree	398(40.3)	218(54.8)	180(45.2)		
Monthly income (RMB)				10.665	0.005
<3000	169(17.8)	83(49.1)	86(50.9)		
3000–3999	301(31.7)	193(64.1)	108(35.9)		
4000–	480(50.5)	269(56.0)	211(44.0)		
Sexual orientation				10.665	0.005
Homosexual	169(17.8)	83(49.1)	86(50.9)		
Bisexual	301(31.7)	193(64.1)	108(35.9)		
Heterosexual/uncertain	480(50.5)	269(56.0)	211(44.0)		
Registered residence				4.337	0.114
Zhejiang	528(53.4)	292(55.3)	236(44.7)		
Other provinces	460(46.6)	272(59.1)	188(40.9)		
Living time in Ningbo (M)				3.910	0.142
<3	169(17.1)	108(63.9)	61(36.1)		
3–24	95(9.6)	52(54.7)	43(45.3)		
24–	724(73.3)	404(55.8)	320(44.2)		
Sample source				6.009	0.014
Online recruitment	529(53.5)	321(60.7)	208(39.3)		
Not online recruitment	459(46.5)	243(52.9)	216(47.1)		

**Table 2:** Cognitive and Behavioral characteristics of HIV-negative men who have sex with men who underwent HIV testing in the past year in Ningbo, China (*N* = 988).

Variables	N (%)	HIV Testing		χ^2^	*P*
		Yes	No		
AIDS awareness				0.429	0.513
No	82(8.3)	44(53.7)	38(46.3)		
Yes	906(91.7)	520(57.4)	386(42.6)		
Cognition of AIDS epidemic among MSM				22.439	<0.001
Nothing serious	285(28.8)	34(37.0)	58(63.0)		
General	611(61.8)	345(56.5)	266(43.5)		
Serious	92(9.3)	185(64.9)	100(35.1)		
Substance use to adjust psychiatric disorders ^#^				23.204	<0.001
No	547(55.4)	275(50.3)	272(49.7)		
Yes	441(44.6)	289(65.5)	152(34.5)		
High-risk sexual behavior ^&^				3.044	0.081
No	495(50.1)	269(54.3)	226(45.7)		
Yes	493(49.9)	295(59.8)	198(40.2)		
Alcohol use before condom-less anal sex				1.541	0.215
No	927(94.2)	524(56.5)	403(43.5)		
Yes	57(5.8)	37(64.9	20(35.1)		
Drug use before condom-less anal sex				-	0.478
No	975(99.2)	554(56.8)	421(43.2)		
Yes	8(0.8)	6(75.0)	2(25.0)		
Group anal sex				0.036	0.849
No	968(98.0)	553(57.1)	415(42.9)		
Yes	20(2.0)	11(55.0)	9(45.0)		
Diagnosed with STD/S in the last three months				5.424	0.020
No	956(96.9)	539(56.4)	417(43.6)		
Yes	31(3.1)	24(77.4)	7(22.6)		
Receiving AIDS interventions ^$^				117.29	<0.001
No	558(56.5)	235(42.3)	320(57.7)		
Yes	430(43.5)	329(76.5)	101(23.5)		

^#^ Substance use to adjust psychiatric disorders meant that MSM had used substances (e.g., cigarettes, alcohol, prescription psychotropic drugs, illegal drugs, etc.) to adjust mental state (e.g., annoyance, nervousness, depression, etc.) in the last three months. ^&^ High-risk sexual behavior meant that MSM had engaged in sexual behaviors without condom use in the past 3 months. MSM who engaged in sexual behaviors with condom use or had not been sexual were defined as non-high-risk behavior. ^$^ Receiving AIDS interventions meant that MSM had received face-to-face AIDS interventions (including AIDS-related knowledge education, HIV testing, and high-risk behavior intervention) provided by medical staff or peers in the last three months.

**Table 3:** Factors associated with the HIV testing in the past year among HIV-negative men who have sex with men in Ningbo, China (*N* = 988).

Variables	Number of HIV Testing %(n/N)	Univariate OR(95%CI)	Multivariate AOR(95%CI)
Sexual orientation			
Homosexual	371(60.9)	1.00	1.00
Bisexual	158(50.5)	0.65(0.50–0.86)	0.57(0.42–0.78)
Heterosexual/uncertain	35(53.0)	0.72(0.44–1.21)	0.65(0.37–1.15)
Substance use to adjust psychiatric disorders			
No	275(50.3)	1.00	1.00
Yes	289(65.5)	1.88(1.45–2.44)	1.39(1.04–1.85)
Receiving AIDS interventions			
No	235(42.3)	1.00	1.00
Yes	329(76.5)	4.44(3.35–5.87)	4.03(3.00–5.42)

**Table 4:** Factors associated with high-risk sexual behaviors among HIV-negative men who have sex with men in Ningbo, China (*N* = 988).

Variables	Number of High-Risk Sexual Behavior % (n/N)	Univariate OR (95%CI)	Multivariate AOR (95%CI)
Marital status			
Single	260(43.2)	1.00	1.00
Married	215(62.1)	2.16(1.65–2.83)	1.72(1.15–2.58)
Divorced/widowed	18(45.0)	1.08(0.57–2.05)	1.18(0.57–2.46)
Education			
Junior high school and below	167(60.5)	1.00	1.00
High school and junior college	159(50.6)	0.67(0.48–0.93)	0.68(0.47–0.97)
College or higher degree	167(42.0)	0.47(0.32–0.65)	0.52(0.35–0.77)
Sexual orientation			
Homosexual	261(42.9)	1.00	1.00
Bisexual	203(64.9)	2.46(1.86–3.26)	2.13(1.54–2.95)
Heterosexual/uncertain	29(43.9)	1.05(0.63–1.74)	1.01 (0.58–1.78)
Receiving AIDS interventions			
No	252(45.2)	1.00	1.00
Yes	241(56.0)	1.54(1.20–1.99)	1.65(1.25–2.20)
